# Detection and molecular characterization of *Blastocystis* sp., *Enterocytozoon bieneusi* and *Giardia duodenalis* in asymptomatic animals in southeastern Iran

**DOI:** 10.1038/s41598-025-90608-w

**Published:** 2025-02-20

**Authors:** Kareem Hatam-Nahavandi, Hanieh Mohammad Rahimi, Mostafa Rezaeian, Ehsan Ahmadpour, Milad Badri, Hamed Mirjalali

**Affiliations:** 1 Department of Parasitology and Mycology, School of Medicine, Iranshahr University of Medical Sciences, Iranshahr, Iran; 2 Foodborne and Waterborne Diseases Research Center, Research Institute for Gastroenterology and Liver Diseases, Shahid Beheshti University of Medical Sciences, Tehran, Iran; 3 Department of Parasitology and Mycology, School of Public Health, Tehran University of Medical Sciences, Tehran, Iran; 4 Department of Parasitology and Mycology, School of Medicine, Tabriz University of Medical Sciences, Tabriz, Iran; 5 Medical Microbiology Research Center, Qazvin University of Medical Sciences, Qazvin, Iran

**Keywords:** *Enterocytozoon bieneusi*, *Giardia duodenalis*, *Blastocystis* sp., *Cryptosporidium* spp., Domestic animals, Iran

## Abstract

Domestic animals can harbor a variety of enteric unicellular eukaryotic parasites (EUEP) with zoonotic potential that pose risks to human health. The aim of this study was to evaluate the occurrence and genetic diversity of EUEP of zoonotic relevance in domestic animals in Iran. Faecal samples were collected from cattle, sheep, camels, goats, donkeys, horse, and dogs. A real-time PCR was performed to detect the parasites, followed by sequence-based genotyping analyses on isolates that tested positive for *Enterocytozoon bieneusi*, *Giardia duodenalis*, and *Blastocystis* sp.. Overall, 53 out of 200 faecal samples (26.5%, 95% CI 20.5–33.2) were positive for one or more EUEP. *Enterocytozoon bieneusi* was found in 23.8%, 12.0%, 26.1%, and 13.3% of cattle, sheep, goats, and camels, respectively. *Giardia duodenalis* was identified in 19.3% of cattle and 6.7% of camels. *Blastocystis* sp. was detected in 5.7% of cattle and 16.7% of camels. *Enterocytozoon bieneusi* genotypes macaque1, J, BEB6, and CHG3 were identified in 3.7% (1/27), 3.7% (1/27), 44.4% (12/27), and 48.2% (13/27) of the isolates, respectively. *Giardia duodenalis* assemblage B and *Blastocystis* subtype 10 were identified in one cattle and one camel isolate, respectively. These findings suggest that domestic animals could serve as potential reservoirs for EUEP of zoonotic relevance and might play a significant role in transmitting these parasites to humans and other animals.

## Introduction

*Giardia duodenalis*, *Blastocystis* sp., *Enterocytozoon bieneusi*, and *Cryptosporidium* spp. are common enteric unicellular eukaryotic parasites (EUEP) that infect humans and other animals^1–3^. *Giardia duodenalis* causes clinical manifestations that vary from asymptomatic cases to acute diarrhea and malabsorption^4^. *Blastocystis* sp. is most frequently found in asymptomatic hosts, and its pathogenicity profile remains unclear^5^. *Cryptosporidium* spp. and *E. bieneusi* can opportunistically cause disseminated infections in immunocompromised individuals such as AIDS patients^6^.

The infections are acquired by ingesting cysts, oocysts, or spores in food or water that has been contaminated by faeces^7,8^. Human infections with *G. duodenalis* can lead to the excretion of up to 2 × 10^5^ cysts per gram of faeces^7^. Infected animals, especially young ones, can shed up to 10^7^ *Cryptosporidium* oocysts and *Giardia* cysts per gram of faeces at the peak of the infection^9^. This demonstrates the significant contribution of livestock to the environmental load of infective (oo)cysts, which can cause waterborne^10^ and foodborne^11^ diarrhea outbreaks in humans worldwide.

Accurate identification of EUEP genotypes requires the use of molecular diagnostic tools, which reveal genetic diversity and zoonotic transmission routes. Techniques such as multilocus sequence typing and PCR-based methods have significantly enhanced our understanding of the prevalence and transmission dynamics of the parasites^3^. These parasites exhibit pronounced intraspecific genetic diversity, reflected in marked differences in host range and specificity, potential for zoonotic transmission, and pathogenicity^2,3,12^. *Giardia duodenalis* encompasses eight genetic assemblages (A-H). Assemblages A and B are commonly identified in humans and other mammalian hosts, whereas C to H are considered to be host-specific, predominantly infecting canids (C and D), ungulates (E), felids (F), rodents (G), and marine pinnipeds (H)^13^. Despite being ‘host-adapted’, assemblages C, D, E, and F have also been reported in humans^14^. Currently, *Cryptosporidium* comprises at least 46 taxonomically valid species, among which, over 20 have been identified in humans^3,15–17^. *Cryptosporidium hominis* and *C. parvum* are responsible for 95% of human cases of cryptosporidiosis, followed by *C. meleagridis, C. felis*, and *C. canis*^15,18^. Eight species have been reported in ungulates, with *C. parvum* and *C. andersoni* being the most prevalent^3,19,20^. Cattle can also carry *C. hominis*, which could be responsible for a number of human *Cryptosporidium* infections^21^. To date, nearly 600 distinct genotypes of *E. bieneusi* have been identified and categorized into 11 major phylogenetic groups, among which 9 zoonotic genotypes (A, BEB4, BEB6, D, EbpA, EbpC, I, J, and Type IV) have been found circulating in domestic animals^22^. Of the 40 identified subtypes of *Blastocystis* sp. (ST1-ST17, ST21, ST23-ST44)^11,23–26^, 12 subtypes (ST1-ST10, ST12, and ST14) have been recognized as zoonotic^27–29^.

Domestic animals harbor a wide variety of EUEP that may be transmitted to humans through faeces. It is therefore essential to understand the occurrence and genetic diversity of these parasites to investigate their zoonotic potential. In Iran, there is a scarcity of data on the molecular epidemiology of EUEP of zoonotic relevance in animal populations, with most of the studies relying on conventional light microscopy for screening, and only a limited number assessing the occurrence and genetic diversity (Table 1). The present study was conducted with the aim of investigating the occurrence, genetic diversity, and zoonotic potential of *Blastocystis* sp., *G. duodenalis*, *E. bieneusi*, and *Cryptosporidium* spp. in various domestic animals in southeastern Iran.

**Table 1:** Frequency and genetic diversity of *Giardia duodenalis*, *Cryptosporidium* spp., *Enterocytozoon bieneusi*, and *Blastocystis* sp. infections reported in domestic animals in Iran, 2008–2024.

Parasite	Host	Sample size (n)	Pos	%	Detection methods	Species/genotype/subtype (n)	References
*Giardia duodenalis*	Cattle	246	23	9.3	CM/PCR	A (4), E (19)	Malekifard and Ahmadpour^30^
*Giardia duodenalis*	Goat	94	15	15.9	CM/PCR	E (15)	Jafari et al.^30^
*Giardia duodenalis*	Goat	100	5	5.0	PCR	E (1)	Kiani-Salmi et al.^31^
*Giardia duodenalis*	Sheep	89	17	19.1	CM/PCR	E (17)	Jafari et al.^30^
*Giardia duodenalis*	Sheep	192	12	6.2	PCR	E (6)	Kiani-Salmi et al.^31^
*Giardia duodenalis*	Horse	42	15	35.7	CM/PCR	A (4), E (9)	Jafari et al.^32^
*Giardia duodenalis*	Dog	315	6	1.9	CM/PCR	A (1), C (3), D (2)	Homayouni et al.^33^
*Giardia duodenalis*	Dog	246	7	2.8	CM/PCR	A (2), C (3), D (2)	Esmailzadeh et al.^34^
*Cryptosporidium* spp.	Cattle	292	35	11.9	CM/PCR	*C. parvum* (35)	Pirestani et al.^35^
*Cryptosporidium* spp.	Cattle	412	78	18.9	CM/PCR	*C. parvum* (8), *C. andersoni* (4)	Fotouhi Ardakani et al.^36^
*Cryptosporidium* spp.	Cattle	272	51	18.7	CM/PCR	*C. parvum* (37), *C. andersoni* (9), *C. bovis* (4)	Keshavarz et al.^37^
*Cryptosporidium* spp.	Cattle	300	85	28.3	CM/PCR	*C. parvum* (45)	Asadpour et al.^38^
*Cryptosporidium* spp.	Cattle	246	55	22.3	CM/PCR	*C. parvum* (50), *C. andersoni* (5)	Mirzai et al.^39^
*Cryptosporidium* spp.	Cattle	217	8	3.7	CM/PCR	*C. parvum* (8)	Mahami Oskouei et al.^40^
*Cryptosporidium* spp.	Cattle	192	9	4.7	PCR	*C. andersoni* (7), *C. bovis* (2)	Firoozi et al.^41^
*Cryptosporidium* spp.	Goat	100	2	2.0	PCR	*C. xiaoi* (2)	Firoozi et al.^41^
*Cryptosporidium* spp.	Sheep	192	11	5.7	PCR	*C. ubiquitum* (5), *C. xiaoi* (6)	Firoozi et al.^41^
*Blastocystis sp.*	Cattle	198	19	9.6	PCR	ST3 (2), ST5 (9), ST6 (2)	Badparva et al.^42^
*Blastocystis* sp.	Cattle	75	25	33.3	CM/PCR	ST5 (9), ST10 (2)	Sharifi et al.^43^
*Blastocystis* sp.	Cattle	32	16	50.0	PCR	ST1 (1), ST5 (1), ST10 (7), ST14 (6)	Mohammad Rahimi et al.^44^
*Blastocystis* sp.	Cattle	40	14	35.0	PCR	ST3 (3), ST10 (7), ST14 (4)	Rostami et al.^45^
*Blastocystis* sp.	Cattle	75	38	50.7	CM/PCR	ST1 (2), ST7 (1), ST10 (1), ST14 (7)	Salehi et al.^46^
*Blastocystis* sp.	Cattle	150	9	6.0	PCR	ST1 (2), ST3 (3), ST10 (4)	Shams et al.^47^
*Blastocystis* sp.	Sheep	150	29	19.3	PCR	ST7 (11), ST10 (18)	Rostami et al.^45^
*Blastocystis* sp.	Sheep	70	30	42.8	PCR	ST5 (2), ST10 (16), ST14 (10)	Mohammad Rahimi et al.^44^
*Blastocystis* sp.	Sheep	100	32	32.0	CM/PCR	ST3 (2), ST5 (2), ST7 (3), ST14 (7)	Salehi et al.^46^
*Blastocystis* sp.	Sheep	150	7	4.7	PCR	ST2 (2), ST3 (2), ST10 (3)	Shams et al.^47^
*Blastocystis* sp.	Camel	150	18	12.0	PCR	ST7 (2), ST10 (9), ST14 (7)	Asghari et al.^48^
*Blastocystis* sp.	Dog	154	29	18.8	CM/PCR	ST2 (8), ST3 (11), ST4 (3), ST7 (3), ST8 (2), ST10 (2)	Mohammadpour et al.^49^
*Enterocytozoon bieneusi*	Cattle	256	48	18.7	PCR	D (22), J (9), M (5)	Kord-Sarkachi et al.^50^
*Enterocytozoon bieneusi*	Cattle	32	11	34.4	PCR	I (7), BEB6 (1), D (2)	Mohammad Rahimi et al.^44^
*Enterocytozoon bieneusi*	Cattle	200	17	8.5	PCR	D (12); J (5)	Shafiee et al.^51^
*Enterocytozoon bieneusi*	Sheep	70	18	25.7	PCR	BEB6 (17)	Mohammad Rahimi et al.^44^
*Enterocytozoon bieneusi*	Horse	26	3	11.5	PCR	BEB6 (1), Horse 1 (1)	Mohammad Rahimi et al.^44^
*Enterocytozoon bieneusi*	Dog	75	4	5.3	PCR	D (4)	Delrobaei et al.^52^

CM: conventional microscopy; PCR: polymerase chain reaction.

## Results

### Microscopic detection of *G. duodenalis* cysts

In the initial examination of the faecal samples using conventional microscopy, 1.5% (3 of 200, 95% CI 0.3–4.3) were found to be positive for *G. duodenalis* cysts, originating from two sheep and one goat.

### Molecular detection of the parasites

Of the 200 faecal samples analyzed, 53 (26.5%, 95% CI 20.5–33.2) tested positive for one or more EUEP, and 147 (73.7%) were negative by qPCR (Tables 2, 3). Single infections were found in 20.5% (41 of 200), and co-infections by two and three parasite species were detected in 5.5% (11 of 200) and 0.5% (1 of 200), respectively (Table 3), resulting in an overall prevalence of 33.0% (66 of 200, 95% CI 26.5–39.9) (Table 2). The overall prevalences of *Blastocystis* sp., *E. bieneusi*, and *G. duodenalis* in animals were 5.0% (95% CI 2.4–9.0), 18.5% (95% CI 13.4–24.6), and 9.5% (95% CI 5.8–14.4), respectively. All analyzed animal faecal samples were negative for *Cryptosporidium* spp.. *Blastocystis* sp. was found in 5.7% of cattle (5 of 88, 95% CI 1.9–12.8) and 16.7% of camels (5 of 30, 95% CI 5.6–34.7). The prevalence rates of *E. bieneusi* were 23.8% (21 of 88, 95% CI 15.4–34.1), 12.0% (6 of 50, 95% CI 4.5–24.3), 26.1% (6 of 23, 95% CI 10.2–48.4), and 13.3% (4 of 30, 95% CI 3.7–30.7), respectively, in cattle, sheep, goats, and camels. *Giardia duodenalis* was identified in 19.3% of cattle (17 of 88, 95% CI 11.7–29.1) and 6.7% of camels (2 of 30, 95% CI 0.8–22.0) (Table 2). Statistical analysis revealed a lack of agreement (*κ*-value: -0.027 [95% CI -0.053 to -0.000]) between microscopy and qPCR for the detection of *G. duodenalis* cysts (Table 4).

**Table 2:** Prevalence of enteric unicellular eukaryotic parasites among domestic animals in Iranshahr by qPCR.

Host/pathogens	*Blastocystis* sp.		*G. duodenalis*		*E. bieneusi*		*Cryptosporidium* spp.		Total	
	Pos. (*n*)	% (95% CI)	Pos. (*n*)	% (95% CI)	Pos. (*n*)	% (95% CI)	Pos. (*n*)	% (95% CI)	Pos. (*n*)	% (95% CI)
Cattle (*n* = 88)	5	5.7 (1.9–12.8)	17	19.3 (11.7–29.1)	21	23.8 (15.4–34.1)	0	0.0	43	48.9 (38.1–59.8)
Sheep (*n* = 50)	0	0.0	0	0.0	6	12.0 (4.5–24.3)	0	0.0	6	12.0 (4.5–24.3)
Goat (*n* = 23)	0	0.0	0	0.0	6	26.1 (10.2–48.4)	0	0.0	6	26.1 (10.2–48.4)
Camel (*n* = 30)	5	16.7 (5.6–34.7)	2	6.7 (0.8–22.0)	4	13.3 (3.7–30.7)	0	0.0	11	36.7 (19.9–56.1)
Donkey (*n* = 5)	0	0.0	0	0.0	0	0.0	0	0.0	0	0.0
Horse (*n* = 1)	0	0.0	0	0.0	0	0.0	0	0.0	0	0.0
Dog (*n* = 3)	0	0.0	0	0.0	0	0.0	0	0.0	0	0.0
Total (*n* = 200)	10	5.0 (2.4–9.0)	19	9.5 (5.8–14.4)	37	18.5 (13.4–24.6)	0	0.0	66	33.0 (26.5–39.9)

**Table 3:** Sequence typing results of the 53 enteric unicellular eukaryotic parasite-positive samples in the present survey.

Sample code	Host	*E. bieneusi*				*G. duodenalis*			*Blastocystis* sp.			Acc No
		qP	NPS	Genotype	Group	qP	NPS	As	qP	NPS	ST	
[1] 12	Camel					+	ns					
[2] 15	Camel	+	+	BEB6	2c							PQ137011
[3] 24	Camel								+	+	ns	
[4] 25	Camel								+	+	ns	
[5] 26	Camel								+	+	ST10	PQ211066
[6] 29	Camel								+	+	ns	
[7] 30	Camel	+	+	Macaque1	6							PQ137025
[8] 31	Camel	+	+	BEB6	2c							PQ137012
[9] 33	Camel								+	+	ns	
[10] 34	Camel	+	+	CHG3	2c							PQ137029
[11] 36	Camel					+	ns					
[12] 40	Cattle	+	+	BEB6	2c							PQ137020
[13] 51	Cattle	+	+	BEB6	2c							PQ137013
[14] 59	Cattle	+	+	CHG3	2c							PQ137030
[15] 63	Cattle	+	ns									
[16] 67	Cattle	+	+	J	2b							PQ137014
[17] 69	Cattle	+	+	BEB6	2c							PQ137026
[18] 70	Cattle	+	+	CHG3	2c							PQ137031
[19] 71	Cattle					+	ns					
[20] 72	Cattle	+	ns									
[21] 73	Cattle					+	ns					
[22] 74	Cattle	+	+	BEB6	2c	+	ns					PQ137027
[23] 75	Cattle	+	+	CHG3	2c	+	ns					PQ137032
[24] 76	Cattle					+	ns					
[25] 78	Cattle	+	+	BEB6	2c	+	ns					PQ137021
[26] 79	Cattle	+	ns			+	ns					
[27] 80	Cattle	+	+	BEB6	2c							PQ137033
[28] 81	Cattle	+	ns			+	ns					
[29] 83	Cattle	+	+	BEB6	2c	+	ns		+	+	ns	PQ137028
[30] 84	Cattle	+	ns			+	ns					
[31] 85	Cattle	+	+	BEB6	2c							PQ137022
[32] 86	Cattle	+	+	BEB6	2c	+	ns					PQ137023
[33] 88	Cattle	+	+	CHG3	2c	+	ns					PQ137024
[34] 89	Cattle					+	ns		+	+	ns	
[35] 93	Cattle	+	+	CHG3	2c				+	+	ns	PQ137015
[36] 97	Cattle					+	+	B	+	+	ns	PQ139658
[37] 99	Cattle								+	ns		
[38] 105	Cattle					+	ns					
[39] 114	Cattle					+	ns					
[40] 116	Cattle					+	ns					
[41] 122	Cattle	+	ns									
[42] 132	Sheep	+	+	CHG3	2c							PQ137016
[43] 133	Sheep	+	ns									
[44] 159	Sheep	+	ns									
[45] 171	Sheep	+	ns									
[46] 173	Sheep	+	+	CHG3	2c							PQ137017
[47] 175	Sheep	+	ns									
[48] 182	Goat	+	+	CHG3	2c							PQ137034
[49] 183	Goat	+	+	CHG3	2c							PQ137008
[50] 185	Goat	+	+	BEB6	2c							PQ137018
[51] 194	Goat	+	+	CHG3	2c							PQ137019
[52] 195	Goat	+	+	CHG3	2c							PQ137009
[53] 196	Goat	+	+	CHG3	2c							PQ137010

qP: qPCR; NPS: nested PCR sequencing; As: assemblage; ST: subtype; ns: not sequenced.

GenBank accession number are provided.

**Table 4:** Measuring the κ coefficient between real-time PCR and conventional microscopy tests for detecting *Giardia duodenalis* in domestic animals.

Test	Real-time PCR		
	Negative	Positive	Total
Conventional microscopy			
Positive	3	0	3
Negative	178	19	197
Total	181	19	200
κ coefficient	-0.0266 (95% CI -0.0531 to -0.0001; SE: 0.0470)		

CI: Confidence Interval; SE: Standard Error of kappa.

### *Enterocytozoon bieneusi* genotyping

**Fig. 1:**
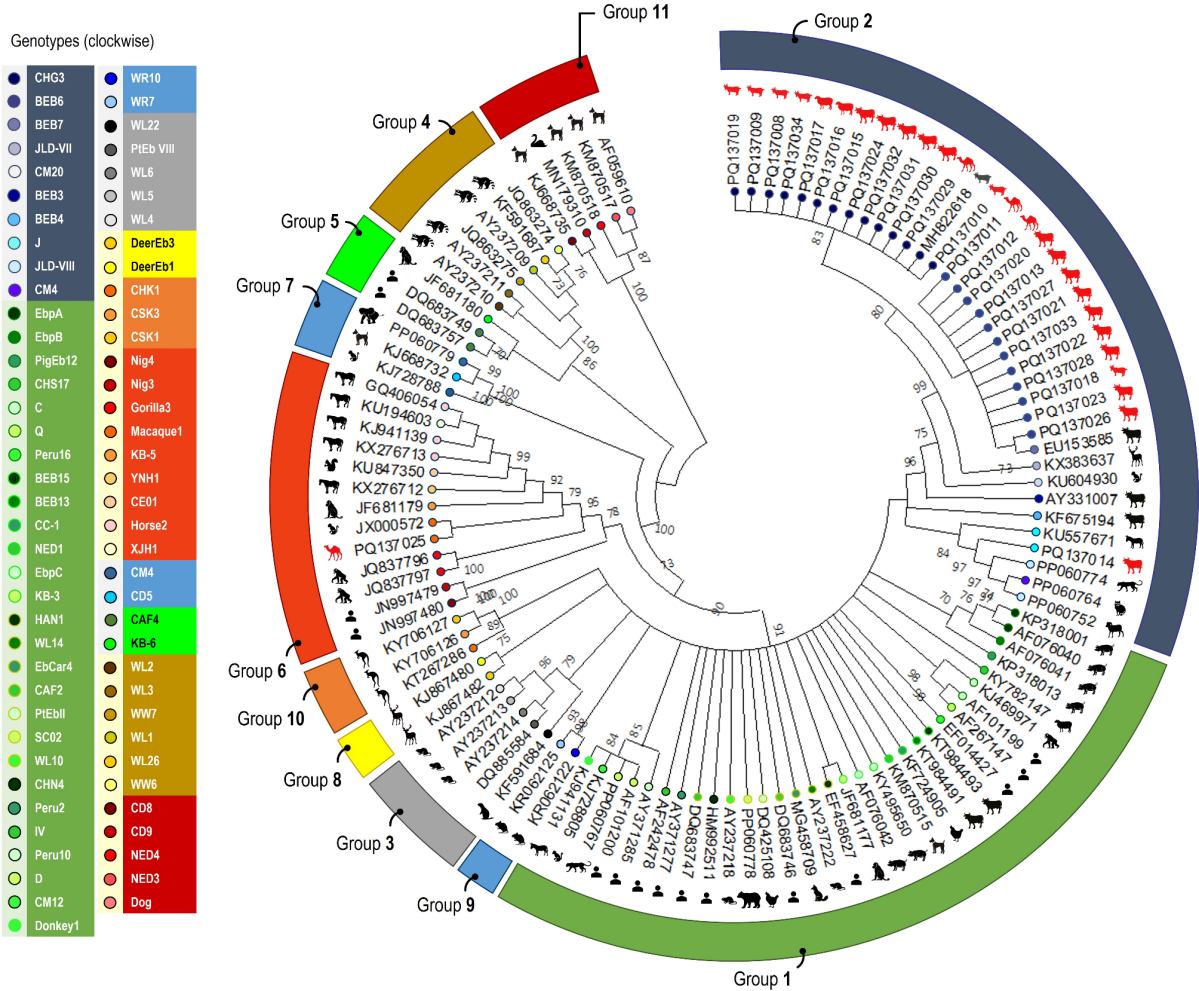
Phylogenetic relationship among *Enterocytozoon bieneusi* genotypes revealed by a maximum likelihood analysis of the partial ITS gene. Numbers on branches are percent bootstrapping values over 70% using 1,000 replicates. The red animal silhouettes indicate the nucleotide sequences generated in the present study. The filled coloured circles indicate the nucleotide sequences of genotypes. Human and animal sequences retrieved from GenBank were included in the analysis for comparative purposes.

Of the 37 faecal DNA samples (4 camels, 21 cattle, 6 goats, 6 sheep) with Ct values ≤ 35, 27 (72.9%) were successfully amplified at the ITS locus using nested-PCR analysis (Table 3). The sequence analysis identified the presence of genotypes J in 3.7% (1 of 27), BEB6 in 44.4% (12/27), CHG3 in 48.2% (13 of 27), and macaque1 in 3.7% (1 of 27) of the samples. The genotype CHG3 was the most common, found in one camel (7.7%), five cattle (38.5%), two sheep (15.4%), and five goats (38.5%). The genotype BEB6 was identified in nine cattle (75.0%), two camels (16.7%), and one goat (8.3%). The genotypes J and macaque1 were each observed in a single sample from cattle and camel, respectively (Table 3). Figure 1 illustrates the phylogenetic tree constructed using representative sequences from the eleven *E. bieneusi* ITS groups and genotypes identified to date. The genotypes CHG3, J, BEB6, and macaque1 were distinctly classified into three genetic groups: 2c, 2b, 2c, and 6, respectively.

### *Blastocystis* sp. subtyping

**Fig. 2:**
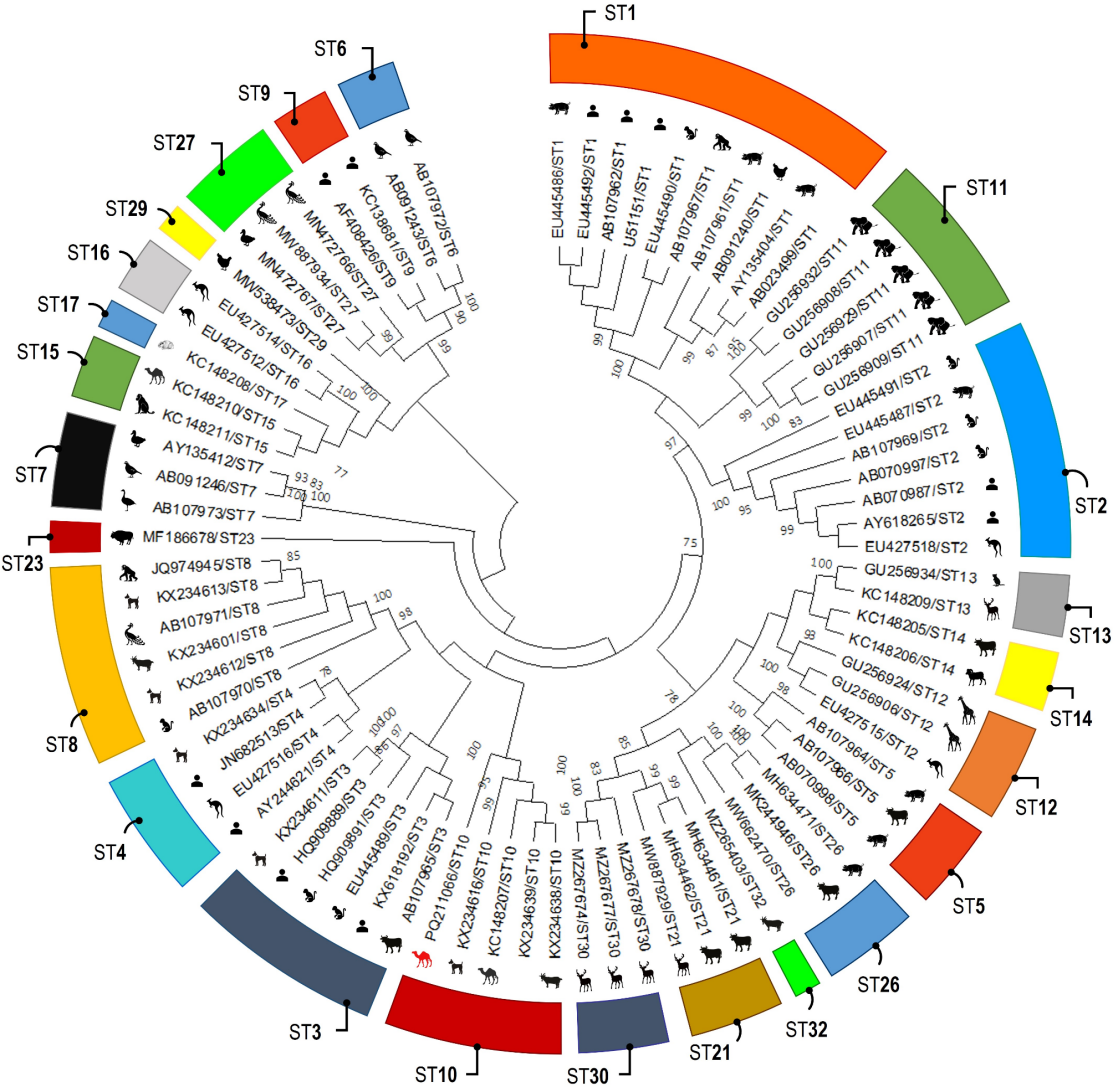
Phylogenetic relationship among *Blastocystis* sp. subtypes revealed by a maximum likelihood analysis of the partial the *ssu* rRNA gene. Numbers on branches are percent bootstrapping values over 70% using 1,000 replicates. The red animal silhouette indicates the nucleotide sequence generated in the present study. Human and animal sequences retrieved from GenBank were included in the analysis for comparative purposes.

Among the 10 faecal DNA samples with Ct values ≤ 35 obtained from 5 camels and 5 cattle, 9 (90.0%) were successfully amplified at the *ssu* rRNA locus using nested-PCR analysis. The sequence analysis identified ST10 in a single camel sample (1 of 9, 11.1%) (Table 3). The phylogenetic analysis revealed a clear distinction among *Blastocystis* sp. subtypes, which matched the classification obtained from BLAST query, in comparison to the reference subtype sequences available in GenBank (Fig. 2). According to homology analysis, the only camel-derived ST10 sequence (PQ211066) shared 100% and 99.82% identities with those from cattle in China (OL981840) and from goats in Colombia (MZ265404), respectively.

### *Giardia duodenalis* genotyping

**Fig. 3:**
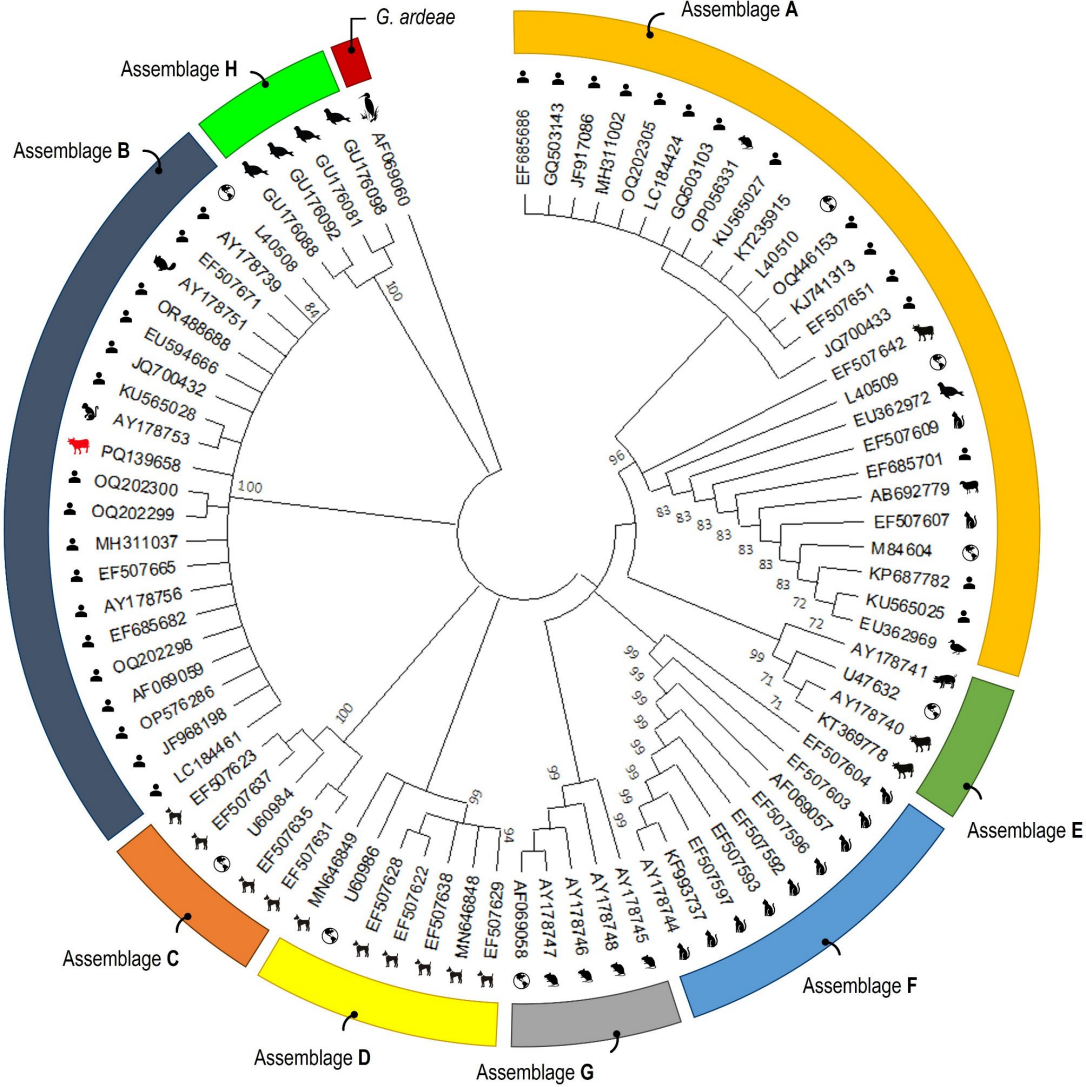
Phylogenetic relationship among *Giardia duodenalis* assemblages revealed by a maximum likelihood analysis of the partial the *gdh* gene. Numbers on branches are percent bootstrapping values over 70% using 1,000 replicates. The red animal silhouette indicates the nucleotide sequence generated in the present study. Human and animal sequences retrieved from GenBank were included in the analysis for comparative purposes.

Among the 19 qPCR-positive samples (2 camels, 17 cattle) analyzed by nested-PCR analysis, one cattle isolate (5.3%) that produced an amplicon of the expected size in *gdh*-PCR was successfully genotyped and classified into zoonotic assemblage B by sequence analysis (Table 3). The phylogenetic analysis consistently grouped the partial nucleotide sequences of the *gdh* locus into *G. duodenalis* assemblages A to H, as well as *G. ardeae*, with strong support indicated by a posterior probability of 100 (Fig. 3).

## Discussion

Domestic animals can be infected with a wide range of parasitic pathogens that can also infect humans. It may not be surprising that around 243 out of the 616 pathogens (39%) known to infect livestock are also capable of infecting humans^53^. Domestic animals with free access to outdoor environments may be at an elevated risk of exposure to pathogens and could serve as neglected reservoirs for zoonotic agents. This study focused on the molecular diversity of *Blastocystis* sp., *E. bieneusi*, and *G. duodenalis* in domestic animals in Iran, particularly assessing their zoonotic potential. There is a lack of molecular data regarding the studied parasite species in animal populations of Iran. The study complements information previously shared by our research group on the epidemiology of gastrointestinal parasites of veterinary health relevance in domestic animal species^54^.

In the present study, 26.5% of the samples tested positive for one or more EUEP using qPCR. In the only comparable molecular-based study on Iranian livestock populations to date, an overall prevalence of 46.2% (80/173) was found in the provinces of Kordestan and Lorestan^44^.

The initial microscopic screening of the samples for *G. duodenalis* cysts revealed an overall prevalence rate of 1.5%, while the subsequent molecular screening detected the parasite at a higher prevalence rate of 9.5%. This difference was predictable, as qPCR demonstrates superior diagnostic performance compared to conventional microscopy due to its higher sensitivity and specificity, enabling the detection of low quantities of cysts and reducing false positives^55^. Furthermore, unlike microscopy, which relies on the technician’s skill and experience for cyst identification—leading to subjective interpretation and variability in results—qPCR provides objective measurements that significantly minimize this variability.

Interestingly, none of the microscopy-positive samples for *G. duodenalis* were detected by qPCR, and the opposite was also true (*κ*-value: -0.027). The difference in diagnostic accuracy between the two screening techniques being compared is supported by the discrepancies noted in previous comparative studies^56,57^. The amount of specimen analyzed may have influenced the results, as the amount of faeces analyzed by microscopy is 100–200 mg. For qPCR, 1.7–2.5 mg of faeces is analysed, assuming that 1 mg equals 1 µL^56^.

Microscopy-based studies have reported *G. duodenalis* prevalences ranging from 0.6% to 40.0% among domestic animal populations in Iran (Table 1). In the only molecular-based study examining the presence of *G. duodenalis* in ruminant animals in Iran, prevalences of 6.2% (12/192), 5.0% (5/100), and 4.2% (8/192) were reported in sheep, goats, and cattle, respectively, in Yazd Province^31^. In the present study, the characterized *G. duodenalis* isolate was identified as belonging to the zoonotic assemblage B. In the study area, a rural lifestyle is predominant, with many households keeping ruminant animals in the courtyards of their mud-walled homes, likely contributing to the cross-transmission of zoonotic assemblage B between animals and humans. This finding contrasts with the existing evidence in the country (Table 1), where assemblages A and E have previously been identified in hoofed animals^30,31,58^, and assemblages A, C, and D in dogs^33,34^. Many studies have shown that assemblage E is the predominant assemblage found in cattle worldwide, followed by assemblage A, and less commonly, assemblage B^14^. Furthermore, three studies have identified assemblages C, D, and F in cattle from the UK, the USA, and Spain^59–61^. In a study on cattle in Scotland^62^, assemblage B was found to be the second most prevalent (18.2%) following assemblage E (77.2%) at the *bg* locus. Remarkably, some assemblage B isolates of bovine origin had 100% sequence identity with a human isolate (KX960128) from Spain^62^. In the current study, the assemblage B isolate was found to have sequence identities of 99.46% and 99.43% with human isolates from Iran (LC184469) and Japan (LC507387), respectively. This suggests that cattle may act as a reservoir for this assemblage, which could have potential public health consequences.

*E. bieneusi* was found in 13.3% of camels, 23.8% of cattle, 12.0% of sheep, and 26.1% of goats, with an overall prevalence rate of 18.5%. *Enterocytozoon bieneusi* infections in livestock populations in Iran have previously been reported in the range of 5.1–34.4% by PCR (Table 1). Molecular studies in different parts of the world have shown that infection rates vary from 2.7% to 91.2%^63–69^. The variations in prevalence rates likely reflect changes in epidemiological scenarios, influenced by differences in parasite genotypes, host populations, environmental conditions, and transmission routes. Our molecular analyses identified the *E. bieneusi* genotypes CHG3, J, BEB6, and macaque1 circulating in the ruminant populations studied. This finding aligns with existing evidence from the country, where *E. bieneusi* genotype BEB6 was previously identified in cattle and sheep from Lorestan and Kordestan Provinces^44^, and genotype J was found in cattle from Ardabil and Fars Provinces^51,70^. The genotypes CHG3 and BEB6 were the most predominant types and displayed a broader animal host range than other genotypes, which aligns with findings from various studies around the world^71–73^. The genotypes CHG3, BEB6, and macaque1 were detected in camels. Molecular investigations of *E. bieneusi* in camelids have thus far been limited to six studies conducted in Algeria, Australia, China, and Peru^74–79^. The presence of zoonotic genotypes in ruminants^70^, along with sporadic infections in humans from ruminant-adapted genotypes (CHG3, J, and BEB6)^80,81^, highlights the significance of these livestock animals in the zoonotic transmission of *E. bieneusi*. Recently, the genotype BEB6 has been identified in raw milk from dairy cattle and sheep^82^, increasing concerns about its potential zoonotic transmission to humans. Several studies have also reported genotype J in various host species, including cattle, yaks, goats, Tibetan sheep, Przewalski’s gazelle, birds, and humans^83–87^.

*Blastocystis* sp. was found in 16.7% of camels and 5.7% of cattle, with an overall prevalence rate of 5.0%. In a recent study conducted in Iran^48^, a prevalence of 12% (18/150) was reported in dromedary and Bactrian camels in Ardabil Province. *Blastocystis* sp. infections in Iranian bovine populations have been previously documented to range from 6.0 to 50.6% using PCR (Table 1). In this study, ST10 was identified in one camel sample, which aligns with existing evidence in Iran, where ST10 was previously detected in 50.0% of camels (9/18) in Ardabil Province^48^. In a recent study conducted among zoo animals in China, 18.2% (2/11) of alpacas were found to be infected with ST10^88^. Although ST10 is commonly isolated from ruminant livestock^45–48^, two recent studies have reported the presence of this animal-specific subtype among human populations from Thailand and Senegal^89,90^, giving the impression of zoonotic transmission.

The study found no *Cryptosporidium* spp. in the animal populations examined, which contrasts with previous reports from Iran. *Cryptosporidium* oocysts were microscopically identified in 1.5% to 63.7% of animal faecal samples (Table 1). The failure to identify *Cryptosporidium* spp. in the current study could be attributed to the small sample sizes, especially for canines and equines.

The main strength of this study is the reporting of molecular epidemiological data on EUEP infections in domestic animals from a geographical region in Iran, where such information was previously lacking. However, the study also has limitations that might have compromised the accuracy of some of the results obtained. First, negative samples examined with direct smear were not subjected to further testing using permanent staining techniques (e.g., trichrome and modified acid-fast staining methods). Second, the sample size for each animal was limited, particularly for canines and equines, which is why we did not find any of the EUEP in these animals.

## Conclusion

This is the first study investigating the molecular epidemiology of EUEP in southeastern Iran. The presence of zoonotic genotypes of *E. bieneusi* (CHG3, BEB6, and J), *G. duodenalis* (assemblage B) and *Blastocystis* sp. (subtype 10) in ruminant animals in this study could contribute to the understanding of the transmission dynamics of these parasites among animals and humans who are in close contact with livestock. However, any conclusions about zoonotic transmission should be drawn with caution, as the presence of similar or different genotypes/subtypes dispersed across various sources does not, by itself, provide conclusive evidence that zoonotic transmission is occurring or not.

## Materials and methods

### Ethics approval and consent to participate

Informed consent was obtained from the owners of the animals involved in this study. Samples were collected during veterinary medical care or checkups. All experimental protocols were approved by the Research Institute for Gastroenterology and Liver Diseases, and all procedures performed in this study adhered to the ethical standards (IR.SBMU.RIGLD.REC.1402.011) set forth by the Ethical Review Committee of the Research Institute for Gastroenterology and Liver Diseases, Shahid Beheshti University of Medical Sciences, Tehran, Iran. Furthermore, all methods were conducted in accordance with relevant guidelines and regulations, and all authors complied with the ARRIVE guidelines.

### Study area

This study was conducted in Iranshahr County, located in the central area of Sistan and Baluchestan province in southeastern Iran^4,54^. The area has a low density of domestic animals, with local families usually raising up to five cattle in a semi-intensive manner in their backyards. Ruminants graze freely in hay fields during the day and are kept at night in small, partially covered structures.

### Sample collection

A total of 200 faecal samples were collected between May and September 2022 from various domestic animal species, including 88 cattle, 55 sheep, 30 camels, 23 goats, 5 donkeys, 3 stray dogs, and one horse. Individual faecal samples (10–20 g of faeces per animal) were taken directly from the rectum of ruminant animals with sterile plastic gloves and placed into 50 mL conical-bottom tubes. Freshly voided faecal samples from canines and equines were collected immediately after defecation from the ground. Faecal samples were excluded from the study if they were collected from the ground and could not be associated with a specific host species. Each faecal sample was given a distinct identification code.

### Microscopic examination

Direct wet mount microscopy was the first method used to detect *Giardia* cysts in faecal samples. All collected samples were then shipped to the Research Institute for Gastroenterology and Liver Diseases, Shahid Beheshti University of Medical Sciences (Tehran) for downstream molecular testing. Faecal samples were stored at 4 °C without preservatives until molecular analysis, for a maximum period of 20 weeks.

### DNA extraction and purification

Total DNA was extracted from an aliquot of approximately 250 mg from each faecal sample using the Stool DNA Extraction Kit (Yekta Tajhiz Azma, Tehran, Iran) following the manufacturer’s instructions^91^. The extracted and purified DNA samples were then stored at -20 °C until molecular analysis.

### Molecular detection

To identify *Blastocystis* sp., *Cryptosporidium* spp., *G. duodenalis* and *E. bieneusi*, a real-time PCR (qPCR) assay was employed to amplify gene fragments of different sizes using specific primers selected for these parasites, as detailed in Table 5^92–95^. This qPCR protocol was conducted on a Rotor Gene Q system (QIAGEN, Germany). The assays were carried out in a total reaction volume of 15 μL, consisting of 7.5 μL of 2X Real-Time PCR Master Mix (BIOFACT, Korea), 0.5 μL of each forward and reverse primer (5 ρmol/μL), 3 μL of template DNA, and 3.5 μL of double distilled water. The cycling profile involved an initial activation step at 95 °C for 10 min, followed by 40 cycles consisting of denaturation at 95 °C for 25 s, annealing at 59 °C for 30 s, and extension at 72 °C for 20 s. Additionally, there was a temperature ramp from 70 °C to 95 °C at a rate of 1 °C per second. Positive and negative controls were included to ensure the validity of the results, and the assays were performed in duplicate to enhance reliability. Post-amplification melting-curve analyses were performed to detect any primer-dimer artifacts and to confirm reaction specificity. Results were regarded as negative if the cycle threshold (Ct) value was greater than 38 or if no amplification curve was observed.

**Table 5:** Primers and annealing temperatures for the identification of *E. bieneusi*, *Cryptosporidium* spp., *Blastocystis* sp., and *G. duodenalis* in this study.

Target species	Gene locus	Primer sequences (5′ to 3′)	Amplicon size (bp)	Annealing (°C)	References
*E. bieneusi*	ITS	F: TGTGTAGGCGTGAGAGTGTATCTG	103	60	Verweij et al.^92^
		R: CATCCAACCATCACGTACCAATC			
*Cryptosporidium* spp.	*ssu* rRNA	F: ATGACGGGTAACGGGGAAT	159	58	Jothikumar et al.^93^
		R: CCAATTACAAAACCAAAAAGTCC			
*Blastocystis* sp.	*ssu* rRNA	F: CGAATGGCTCATTATATC	220	60	Mohammad Rahimi et al.^94^
		R: AGTTAAGCTGATAGGGCAGAAACT			
*G. duodenalis*	*gdh*	F: GCTTCCACCCCTCTGTCAA	214	60	Bahramdoost et al.^95^
		R: CCRGCAGGAACGTCRGTGT			

ITS: internal transcribed spacer; *ssu* rRNA: small subunit ribosomal RNA; *gdh*: glutamate dehydrogenase.

### Genetic characterization

Samples with Ct values of 35 or lower were re-evaluated through sequence-based genotyping analyses utilizing specific markers to assess the molecular diversity of *Blastocystis* sp., *E. bieneusi*, and *G. duodenalis*. A direct PCR protocol targeting a 600-bp partial sequence of the *ssu* rRNA gene of *Blastocystis* sp. was conducted as described by Scicluna et al.^96^. A 410-bp fragment of the ITS gene of *E. bieneusi* was amplified using a previously described nested PCR protocol^97^. A nested-PCR protocol was employed to amplify the glutamate dehydrogenase (*gdh*) gene of *G. duodenalis*, resulting in a final PCR product of 530 bp^98^.

### Sequence analyses

The PCR products of the expected size were directly sequenced in both directions using the internal primer sets described above on an ABI 3130 automated DNA sequencer (Applied Biosystems, USA). The results of the sequencing were refined and trimmed using BioEdit software version 7.2.6.1. The Basic Local Alignment Search Tool (BLAST) (http://www.ncbi.nlm.nih.gov/blast/) was employed to compare the generated nucleotide consensus sequences with reference sequences available in GenBank. All sequences obtained in this study have been deposited in the GenBank database under the following accession numbers: PQ137008 to PQ137034 (*E. bieneusi*), PQ211066 (*Blastocystis* sp.) and PQ139658 (*G. duodenalis*).

### Phylogenetic analyses

Nucleotide sequences generated in this study, along with relevant reference sequences retrieved from GenBank, were used to construct phylogenetic trees. The neighbor-joining method was employed to assess the phylogenetic relationships among genotypes and subtypes, with genetic distances calculated with the Jukes-Cantor model. Branch reliability was evaluated through bootstrapping with 1,000 replicates. Phylogenetic analyses were conducted using MEGA XI software^99^.

### Statistical analysis

Cohen’s kappa coefficient (*κ*) was used to evaluate the agreement between microscopy and qPCR in detecting *Giardia* infection, with interpretations ranging from no agreement (*κ* ≤ 0) to almost perfect agreement (0.81 < *κ* ≤ 1). Data were analyzed using SPSS version 27 (IBM, Armonk, NY, USA).

## Data Availability

All sequences obtained in this study have been deposited in the GenBank database (https://www.ncbi.nlm.nih.gov/nucleotide/) under the following accession numbers: PQ137008 to PQ137034 (*E. bieneusi*), PQ211066 (*Blastocystis* sp.) and PQ139658 (*G. duodenalis*).
